# Metabolic Profiles of Serum and Ovarian Tissue in Taihe Black-Boned Silky Fowl During the Early and Peak Laying Periods

**DOI:** 10.3390/ani15070912

**Published:** 2025-03-22

**Authors:** Xuan Huang, Shibao Li, Zhaozheng Yin

**Affiliations:** Animal Science College, Zijingang Campus, Zhejiang University, Hangzhou 310058, China22317074@zju.edu.cn (S.L.)

**Keywords:** metabolomics, serum, ovary

## Abstract

Egg production is a complex biological process closely linked to ovarian development and metabolic adaptation in laying hens. As the core reproductive organ, the ovary undergoes significant changes during different egg-laying stages. This study employed untargeted metabolomics to analyze metabolites in serum and ovarian tissues of hens at 20W and 30W. The results revealed that metabolic reprogramming in ovarian tissues was more pronounced than in serum. Shared metabolites between serum and ovarian tissues demonstrated coordinated interactions between systemic and local metabolic networks. The synthesis of prostaglandin E1 during lipid metabolism was identified as a key driver of ovulation and hormone production. Extracellular matrix remodeling and polyamine metabolism, particularly spermidine/spermine, enhanced cell adhesion and antioxidant capacity during ovarian development. These findings provide new insights into follicular development, ovulation regulation, and steroid hormone biosynthesis, while suggesting potential metabolic targets to improve poultry reproductive efficiency.

## Simple Summary

The ovary is a crucial organ for egg production in poultry, but the specific mechanisms underlying ovarian development during different laying stages remain unclear. In this study, metabolomics technology was used to analyze the changes in metabolites in the blood and ovaries of hens during the early laying period (20W) and peak laying period (30W), identifying key factors associated with ovarian development and reproductive efficiency.

## 1. Introduction

Eggs are an important source of high-quality protein in the human diet, and the avian ovary, as the key organ responsible for egg production, undergoes dynamic changes during its development that play a pivotal role in determining egg-laying performance. The chicken ovary begins rapid growth at approximately 14 weeks of age and reaches sexual maturity between 18 and 20 weeks [1,2]. After sexual maturity, chickens enter the laying period, which is typically divided into the early laying stage, peak laying stage, and late laying stage [3]. Once chickens begin laying eggs, their egg-laying performance rapidly reaches its peak within one to two weeks, maintains a high laying rate for several months, and then gradually declines into the late laying stage [4]. In recent years, an increasing number of studies have focused on the ovarian changes during different laying stages. For example, Hu et al. [5] used transcriptomic analysis to study the ovaries of black Muscovy ducks during the early, peak, and late laying stages, identifying potential regulatory genes and candidate pathways for each stage. A recent study combined transcriptomics with chromatin accessibility analysis to explore the molecular mechanisms of follicular development in chicken ovaries across different laying stages [6]. However, current research primarily focuses on the transcriptional level, leaving a significant gap in understanding the molecular processes that ultimately lead to ovarian phenotypes.

Metabolomics is a frontier omics technology that has emerged following transcriptomics and proteomics. It systematically analyzes small-molecule metabolites (molecular weight < 1500 Da) within organisms, revealing their types, abundance, and dynamic changes [7,8]. As the omics approach most directly reflecting phenotypic states, metabolomics can comprehensively uncover a large number of metabolites in the body, providing unique insights into biological processes. In recent years, metabolomics has been widely applied in livestock and poultry reproduction research. For instance, Pan et al. [9] used untargeted metabolomics to investigate the dynamic changes in pig ovaries during different developmental stages and pregnancy. Yin et al. [10] through untargeted metabolomics, discovered that dietary energy levels regulate ovarian development during sexual maturation in chickens by influencing energy metabolism, fatty acid synthesis, and steroid hormone biosynthesis. However, no studies have yet reported on the metabolic profiles of chicken ovarian development during different laying stages.

The Taihe black-boned silky fowl (TBSF), an indigenous breed from Jiangxi Province, China, is a genetically distinct resource valued for its dual role in food production and traditional Chinese medicine. Historically documented in traditional Chinese medicine literature, this breed is renowned for medicinal properties including immune modulation, fatigue alleviation, and therapeutic effects on diabetes, anemia, and gynecological disorders, such as menstrual irregularities and postpartum complications [11]. Recent research further highlights bioactive peptides derived from protein hydrolysates of this breed as potential agents for osteoporosis prevention [12]. Despite its medicinal and economic significance, the reproductive performance of TBSF remains suboptimal, characterized by low egg-laying rates and a brief peak production period. The transition from early laying stage to peak egg production represents a critical phase in poultry ovarian maturation and reproductive efficiency. During this period, physiological processes, such as follicular development, steroid hormone synthesis, and metabolic reprogramming, are closely tied to egg-laying performance. However, the metabolic mechanisms governing this transition, particularly in indigenous breeds, like TBSF, remain poorly understood, limiting efforts to improve their productivity through targeted interventions.

This study aimed to investigate the metabolic characteristics of serum and ovarian tissues in TBSF during the early and peak laying stages. By employing untargeted metabolomics, we longitudinally tracked the metabolic dynamics during the transition from the early laying stage to the peak laying stage. We aimed to identify key metabolites and pathways associated with physiological adaptations occurring in these critical stages. Our findings are expected to provide new insights into the metabolic mechanisms driving ovarian development and egg-laying performance and offer a novel perspective on the stage-specific regulation of egg production during ovarian development.

## 2. Materials and Methods

### 2.1. Ethics Approval

All experimental procedures involving animals were performed following the ARRIVE recommendations. The protocol was reviewed and approved by the Animal Experimentation Ethics Committee of Zhejiang University (Ethics Approval No. ZJU20190149), ensuring strict compliance with China’s national standards for laboratory animal welfare throughout the study.

### 2.2. Animal Feeding and Sample Collection

All hens were transferred to individual cages for feeding at 100 days of age and gradually adapted to the cage environment between 100 and 120 days of age. Starting from 112 days of age (16 weeks), a daily photoperiod of 13 h was implemented, with an increase of 1 h per week until reaching 16 h of light/8 h of darkness at 140 days of age (20 weeks) to induce the onset of the laying period. This photoperiod regimen was maintained consistently throughout the laying phase to ensure stability of the light cycle.

The recording and statistical methods for egg production followed our previous study [13], with recorded indicators including daily egg count and laying rate. At 140 days of age (20 weeks), the laying rate of TBSF was approximately 2%. The sampling time during the peak laying period was set at 210 days of age (30 weeks), by which time the flock’s laying rate had stabilized above 60%.

Blood and ovarian tissue samples were collected from the same individuals of TBSF during the early laying period (*n* = 6) and peak laying period (*n* = 6). Sampling was conducted in the afternoon when most hens had completed laying for the day, minimizing the impact of hormone fluctuations during the laying process on experimental results. For serum collection, blood was drawn from the wing veins using vacuum blood collection tubes without anticoagulant. The whole blood was allowed to clot at room temperature for 30 min and then centrifuged at 3000 rpm for 10 min at 4 °C. The supernatant serum was carefully aspirated, aliquoted into sterile EP tubes, flash-frozen in liquid nitrogen, and stored at −80 °C for subsequent non-targeted metabolomics (LC-MS platform) and hormone detection.

For ovarian tissue collection, hens were euthanized by cervical dislocation, and the ovaries were rapidly excised from the abdominal cavity. The ovarian tissues were rinsed three times with pre-chilled phosphate-buffered saline (PBS) to remove atretic follicles, hierarchical follicles (F1–F5), and postovulatory follicles. The remaining ovarian tissues (primarily ovarian stroma and small undifferentiated follicles) were placed in a pre-chilled sterile Petri dish, minced into approximately 1 mm^3^ fragments using ophthalmic scissors, aliquoted into 2 mL RNase-free cryotubes, flash-frozen in liquid nitrogen for 30 min, and subsequently stored at −80 °C for long-term preservation. All serum and ovarian tissue samples were derived from the same individuals to ensure consistency and comparability in the analysis.

### 2.3. Serum Hormone Measurement

Serum levels of follicle-stimulating hormone (FSH), luteinizing hormone (LH), estradiol (E2), prolactin (PRL), and progesterone (P4) in chickens were quantified using ELISA kits (Jiangsu MEIMIAN Bio; catalog numbers: 627, 12062, 7720, 622, 9556). The detection ranges and sensitivities for each hormone were as follows: E2, 2–64 pmol/L with a sensitivity of <1.0 pmol/L; LH, 0.1–12 ng/mL with a sensitivity of <0.1 ng/mL; FSH, 0.1–24 mIU/mL with a sensitivity of <0.1 mIU/mL; PRL, 1.0–40 ng/mL with a sensitivity of <1.0 ng/mL; and P4, 0.1–16 nmol/L with a sensitivity of <0.1 nmol/L. The intra-assay and inter-assay coefficients of variation (CVs) for all measurements were less than 15%.

### 2.4. Metabolite Extraction, Detection, and Analysis

Serum and ovarian tissue samples were subjected to non-targeted metabolomics analysis by Novogene Co., Ltd. (Tianjin, China). Ovarian tissues were ground in liquid nitrogen, homogenized in pre-chilled 80% methanol (4 °C), vortexed, ultrasonicated in an ice bath, and centrifuged (12,000× *g*, 15 min, 4 °C) to collect the supernatant. Serum samples were directly mixed with 80% methanol (containing 0.1% formic acid) and centrifuged (12,000× *g*, 10 min, 4 °C). All supernatants were diluted with MS-grade water to 53% methanol, filtered through 0.22 μm nylon membranes, and analyzed using a liquid chromatography-tandem mass spectrometry (LC-MS/MS) system (UltiMate 3000 (ThermoFisher, Waltham, MA, USA) coupled with Q Exactive HF-X (ThermoFisher, Waltham, MA, USA), HSS T3 column, gradient elution protocol as described by Want et al. [14]). Quality control included pooled QC samples and blank controls, processed identically to experimental samples.

UHPLC-MS/MS data were analyzed using Compound Discoverer 3.1 (CD3.1, ThermoFisher, Waltham, MA, USA) for metabolite identification. Metabolites were annotated using three databases: Kyoto Encyclopedia of Genes and Genomes (KEGG) (https://www.genome.jp/kegg/pathway.html, accessed on 15 June 2024), the Human Metabolome Database (HMDB) (https://www.hmdb.ca/metabolites, accessed on 15 June 2024), and LIPID MAPS (https://www.lipidmaps.org/, accessed on 15 June 2024). Principal component analysis (PCA) and partial least squares discriminant analysis (PLS-DA) were performed using metaX software (Version 2.0.0) [15]. Differential metabolites were identified based on a variable influence on projection (VIP) score > 1, *p* < 0.05, and fold change (FC) >1.5 or <0.67. KEGG pathway enrichment and graphical representation of metabolites were performed using the ggplot2 (Version 3.4.4) package in R (Version 4.3.1).

### 2.5. Statistical Analysis

Quantitative data derived from follicular counts and hormone assays were presented as mean ± standard error of the mean (SEM). Statistical analyses were performed using SPSS (version 26.0, IB Corp., Armonk, NY, USA), with between-group comparisons conducted through independent samples *t*-tests. Visualization of experimental results was generated using GraphPad Prism (version 8.0, GraphPad Software Inc., San Diego, CA, USA).

## 3. Results

### 3.1. Follicle Counts and Serum Hormone Levels

**Figure 1:**
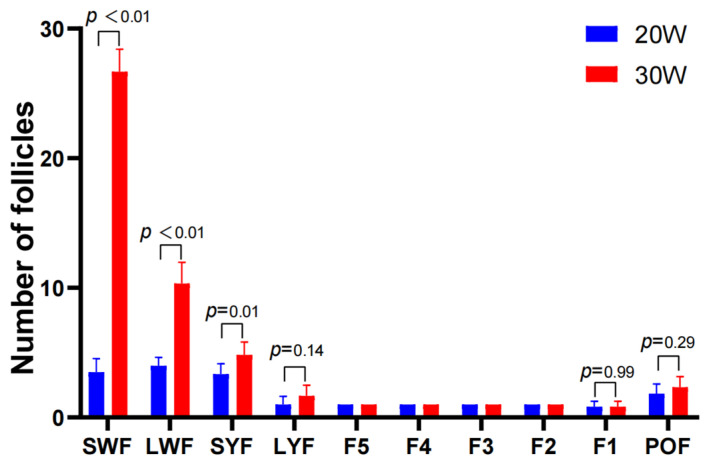
Follicle number of TBSF at 20W and 30W.

The follicle statistics illustrate the number changes of follicles at different developmental stages in TBSF at 20 weeks and 30 weeks of age. The results showed that the number of pre-hierarchical small white follicles (SWF), large white follicles (LWF), and small yellow follicles (SYF) significantly increased from the early laying period (20 weeks) to the peak laying period (30 weeks) (Figure 1). Therefore, in the metabolomic analysis of ovarian tissues, we primarily focused on pre-hierarchical follicles and ovarian stroma as the main research targets, excluding hierarchical follicles (F1–F5), atretic follicles, and postovulatory follicle (POF).

**Figure 2:**
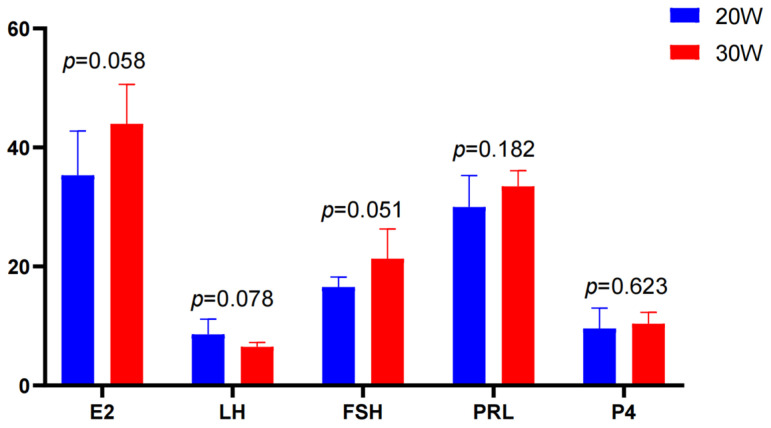
Comparison of serum hormone levels between 20W and 30W groups. E2: Estradiol (pg/mL); LH: Luteinizing hormone (mIU/mL); FSH: Follicle-stimulating hormone (mIU/mL); PRL: Prolactin (ng/mL); PROG: P4 (ng/mL).

The hormone results showed that there were different trends in serum hormone levels between the 20W group and the 30W group. The level of luteinizing hormone (LH) was slightly higher at 20 weeks of age than at 30 weeks of age. The levels of estradiol (E2) and follicle-stimulating hormone (FSH) in the 30W group were higher than those at 20 weeks of age, while the levels of prolactin (PRL) and progesterone (P4) showed minimal variation between the two groups and maintained a relatively stable trend. Overall, the hormone levels in the 30-week group were slightly higher than those in the 20W group, with particularly pronounced increases observed for E2 and FSH (Figure 2).

### 3.2. Metabolomics Quality Control

**Figure 3:**
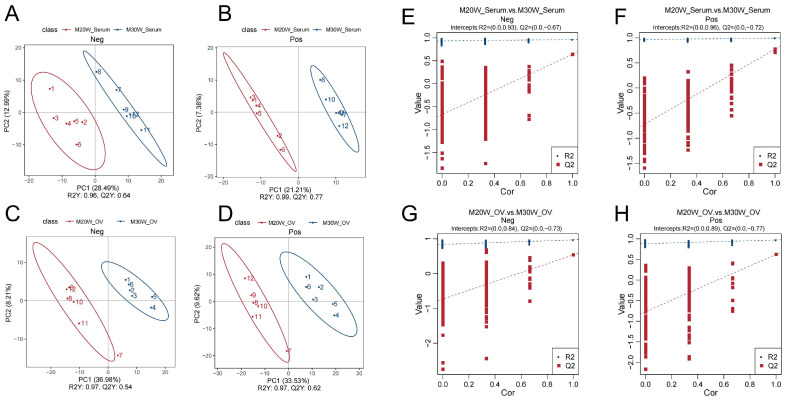
PLS-DA score plot and validation plot of differential metabolites. (**A**) PLS-DA score plot showing the separation of metabolites in the negative ion mode in serum; (**B**) PLS-DA score plot showing the separation of metabolites in the positive ion mode in serum; (**C**) PLS-DA score plot showing the separation of metabolites in the negative ion mode in ovary samples; (**D**) PLS-DA score plot showing the separation of metabolites in the positive ion mode in ovary samples; (**E**) Permutation test of PLS-DA in the negative ion mode for serum samples; (**F**) Permutation test of PLS-DA in the positive ion mode for serum samples; (**G**) Permutation test of PLS-DA in the negative ion mode for ovary samples; (**H**) Permutation test of PLS-DA in the positive ion mode for ovary samples.

The Pearson correlation coefficients (R^2^) for serum metabolome samples ranged from 0.986 to 0.996 (Supplementary Figure S1A,B), while those for ovarian tissue metabolome samples ranged from 0.990 to 0.993 (Supplementary Figure S1C,D). The Pearson correlation coefficients between quality control samples demonstrated high reproducibility and reliability of the serum and ovarian tissue metabolomics data. To identify differential metabolites, we sequentially employed principal component analysis (PCA), partial least squares discriminant analysis (PLS-DA), and cross-validation. PCA results revealed significant separation of samples from different groups in both serum and ovarian tissue metabolomics within the metabolome (Supplementary Figure S2A–D). PLS-DA further confirmed distinct metabolic characteristics among groups (Figure 3A–D). In cross-validation, R2Y represents the explanatory power of the model, while Q2Y indicates its predictive capability. Both R2Y and Q2Y values exceeded 0.5, demonstrating robust cumulative explanatory and predictive performance. To assess potential overfitting, permutation tests were conducted. The observation that the blue line (R2) consistently remained above the red line (Q2) suggested a low risk of model overfitting (Figure 3E–H).

### 3.3. Correlation Analysis Between Serum Metabolites and Ovarian Tissue Metabolome

**Figure 4:**
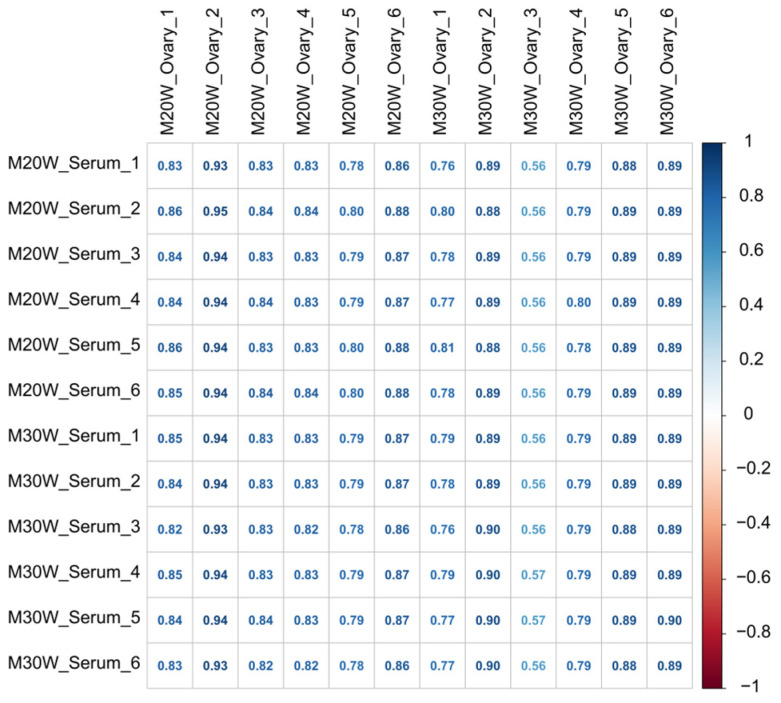
Correlation heatmap of serum and ovarian tissue metabolites.

To explore the potential relationship between serum metabolites and the ovarian tissue metabolome, a correlation analysis was conducted, and the results are presented in Figure 4. The heatmap revealed a significant correlation between serum metabolites and ovarian tissue metabolites. Most metabolites exhibited strong positive correlations, with correlation coefficients ranging from 0.56 to 0.95. Notably, in the 20W and 30W groups, some samples’ metabolites showed exceptionally high correlation coefficients of up to 0.95. Meanwhile, a few samples displayed relatively weaker correlations, with the lowest observed correlation coefficient being 0.56. Additionally, differences in correlation patterns were noted across different groups, suggesting that serum metabolic characteristics may be closely linked to ovarian metabolic status. These findings provide valuable data for further investigation into the metabolic regulatory mechanisms underlying reproductive processes.

### 3.4. Metabolite Annotation

Metabolite annotation based on the KEGG, HMDB, and LIPID Maps databases revealed distinct features in biological pathways and molecular categories of metabolites in serum and ovarian tissues. Serum metabolites annotated by KEGG were primarily associated with lipid metabolism, amino acid metabolism, and carbohydrate metabolism (Supplementary Figure S3A,B). HMDB classification showed the most abundant categories as lipids and lipid-like molecules, organic acids and derivatives, and organoheterocyclic compounds (Supplementary Figure S3C,D). Further classification of serum lipid molecules via LIPID Maps indicated that glycerophosphocholines, fatty acids and conjugates, and glycerophosphoethanolamines were the most enriched classes (Supplementary Figure S3E,F). In ovarian tissues, KEGG annotation similarly highlighted lipid metabolism and amino acid metabolism, along with metabolism of cofactors and vitamins (Supplementary Figure S4A,B). HMDB classification aligned with serum results, dominated by lipids and lipid-like molecules, organic acids and derivatives, and organoheterocyclic compounds (Supplementary Figure S4C,D). LIPID Maps analysis further demonstrated that ovarian lipid molecules were predominantly enriched in glycerophosphocholines, fatty acids and conjugates, and steroids (Supplementary Figure S4E,F).

### 3.5. Differential Expressed Metabolites (DEMs) Characteristic

**Figure 5:**
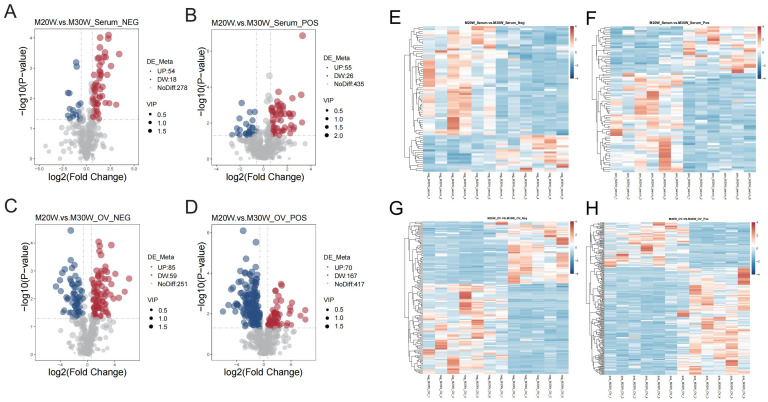
Volcano plot and heatmap of differential metabolites. (**A**) Volcano plot showing differential metabolites in negative ion mode in serum. Red represents upregulated metabolites, blue represents downregulated metabolites; (**B**) Volcano plot showing differential metabolites in positive ion mode in serum; (**C**) Volcano plot showing differential metabolites in negative ion mode in ovary samples; (**D**) Volcano plot showing differential metabolites in positive ion mode in ovary samples; (**E**) Heatmap of the differential metabolites in serum for the negative ion mode; (**F**) Heatmap of the differential metabolites in serum for the positive ion mode; (**G**) Heatmap of the differential metabolites in ovary samples for the negative ion mode; (**H**) Heatmap of the differential metabolites in ovary samples for the positive ion mode.

**Figure 6:**
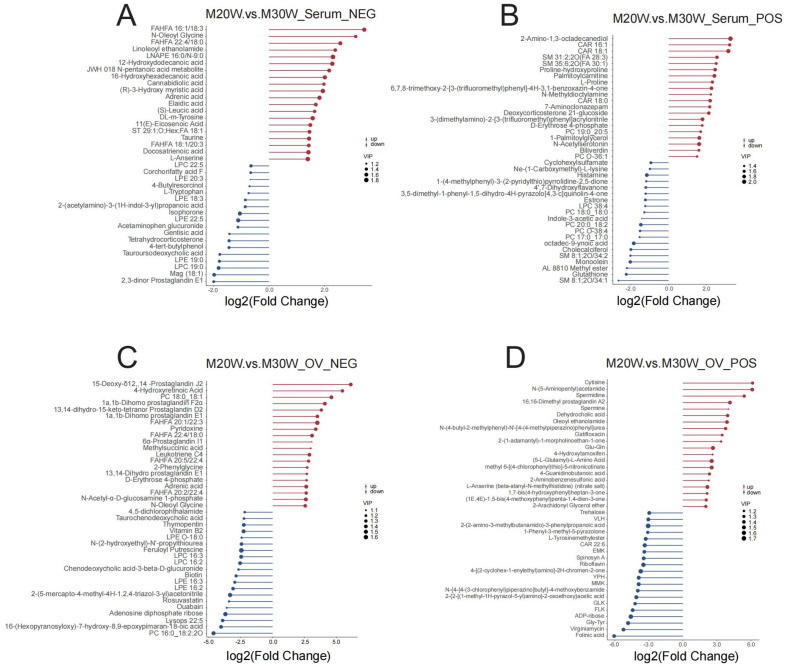
Differential metabolites in stick bar plot. (**A**) Differential metabolites in negative ion mode in serum; (**B**) Differential metabolites in positive ion mode in serum; (**C**) Differential metabolites in negative ion mode in ovary; (**D**) Differential metabolites in positive ion mode in ovary.

**Figure 7:**
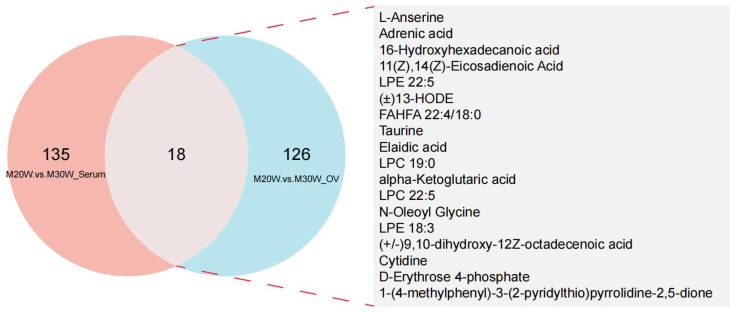
Venn diagram of differential metabolites between serum and ovary. M20W vs. M30W Serum: Differential metabolites in serum between 20W and 30W groups; M20W vs. M30W Ovary: Differential metabolites in ovary between 20W and 30W groups; Overlap: Shared differential metabolites between serum and ovary samples.

A total of 816 metabolites were identified in the serum metabolome (negative ion mode: 350; positive ion mode: 516). Using thresholds of variable importance in projection (VIP) > 1.0, fold change (FC) > 1.5 or FC < 0.667 (*p* < 0.05), 72 and 81 significant differential metabolites were detected in negative and positive ion modes, respectively (*p* < 0.05). Among these, 54 metabolites were significantly upregulated and 18 downregulated in negative ion mode (Figure 5A), while 55 were upregulated and 26 downregulated in positive ion mode (Figure 5B; Supplementary Table S1). In the ovarian tissue metabolome, 1049 metabolites were identified (negative ion mode: 395; positive ion mode: 654). Screening revealed 144 and 237 significant differential metabolites in negative and positive ion modes, respectively (*p* < 0.05). Specifically, 55 metabolites were upregulated and 90 downregulated in the negative ion mode (Figure 5C), whereas 70 were upregulated and 167 downregulated in the positive ion mode (Figure 5D; Supplementary Table S2). To investigate the differences in metabolites between groups, we constructed heatmaps of all DEMs in serum and ovarian tissues. The heatmaps illustrate the relative abundance of metabolites using a color gradient, with red indicating high abundance and blue indicating low abundance. Cluster analysis revealed distinct segregation patterns of DEMs across groups (Figure 5E–H). In addition, we presented the top 20 upregulated and downregulated differential metabolites in the serum metabolome and ovarian tissue metabolome under positive and negative ion modes. The main metabolites in serum included fatty acid derivatives, amino acids, and compounds related to energy metabolism (Figure 6A,B). In the ovary, the primary metabolites consisted of lipids, amino acids, and metabolites involved in steroid hormone synthesis (Figure 6C,D). The serum and ovarian tissue metabolomes shared 18 common metabolites, which are present in both serum and ovarian tissues. These metabolites may indicate their critical roles in systemic regulation and localized ovarian metabolism (Figure 7).

### 3.6. Correlation Analysis of Differential Metabolites

**Figure 8:**
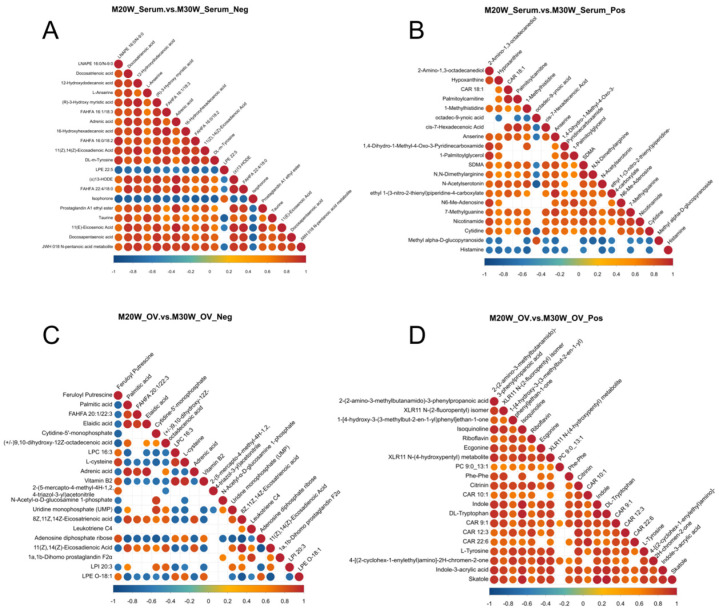
Correlation analysis of differential metabolites. (**A**) Correlation analysis of differential metabolites in negative ion mode in serum; (**B**) Correlation analysis of differential metabolites in positive ion mode in serum; (**C**) Correlation analysis of differential metabolites in negative ion mode in ovarian samples; (**D**) Correlation analysis of differential metabolites in positive ion mode in ovarian samples.

The interactions of metabolites in serum and ovarian tissues at 20 weeks and 30 weeks were analyzed through a metabolite interaction network. The results showed that in the negative ion mode of serum (Figure 8A), there was a strong positive correlation between lipid-related and amino acid-related metabolites, suggesting that these metabolites may have a synergistic role in serum metabolism. In positive ion mode (Figure 8B), more complex metabolite interactions were observed, which may be closely related to the regulation of other metabolic networks in serum. In ovarian tissues, negative ion mode (Figure 8C) revealed significant correlations among lipid-related metabolites, indicating the important role of lipid metabolism in ovarian development and function. In positive ion mode (Figure 8D), certain metabolites were found to be associated with the regulation of ovarian function, suggesting that these metabolites may play a role in controlling ovarian activity.

The changes in correlations among these metabolites provide important molecular markers for a deeper understanding of ovarian development, hormone regulation, and metabolic dynamics. They also offer valuable insights into the metabolic mechanisms in the ovary and serum.

### 3.7. DEMs Functional Enrichment

**Figure 9:**
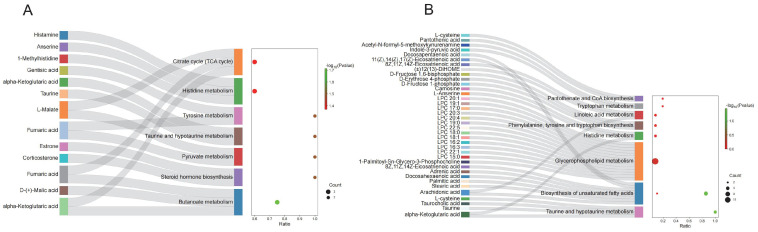
KEGG pathway enrichment of differential metabolites. (**A**) KEGG pathway enrichment of differential metabolites in serum; (**B**) KEGG pathway enrichment of differential metabolites in ovary tissue.

To explore the potential biological functions of DEMs, we conducted KEGG pathway enrichment analysis on DEMs from serum and ovarian tissues. The results indicated that serum DEMs were predominantly enriched in butanoate metabolism, tyrosine metabolism, taurine and hypotaurine metabolism, pyruvate metabolism, and the citrate cycle (Figure 9A; Supplementary Table S3). In ovarian tissues, DEMs were mainly associated with taurine and hypotaurine metabolism, GnRH signaling pathway, and biosynthesis of unsaturated fatty acids (Figure 9B; Supplementary Table S4).

## 4. Discussion

In this study, a non-targeted metabolomics approach was employed to comparatively analyze metabolites in serum and ovarian tissues of poultry during 20W and 30W. Focusing on two critical stages of dynamic changes in egg-laying performance, we aimed to delve into potential candidate metabolites physiologically associated with ovarian development, thereby providing metabolic candidate biomarkers and metabolic pathways to elucidate avian ovarian development and related reproductive processes.

### 4.1. Spatiotemporal Heterogeneity in Systemic and Local Metabolic Regulation

Serum metabolites predominantly reflect global metabolic status [16], whereas ovarian tissue metabolites delineate compartmentalized functional dynamics [17]. This study revealed more pronounced metabolic alterations in ovarian tissue compared to serum, indicating that the ovarian microenvironment undergoes substantial metabolic reprogramming during critical developmental transitions. Notably, shared metabolites between these compartments unveiled a cooperative interplay between systemic and localized metabolic networks. These findings further suggest that ovarian metabolic states are governed not only by microenvironment-specific regulatory precision but also sustained through integration with organism-wide metabolic circuits.

### 4.2. Lipid Metabolic Dynamics Drive Ovulation and Hormone Synthesis

In chicken ovaries, from the initial to peak egg-laying stages, significant lipid metabolic reprogramming may coordinate ovarian development and ovulation through prostaglandin synthesis and lipid signaling [18,19]. The upregulation of FAHFA 16:0/18:2 in serum suggests enhanced systemic lipid mobilization. These long-chain fatty acids are likely transported to the ovaries via serum albumin binding and locally converted to arachidonic acid (upregulated in ovary tissues) [20,21]. As a critical precursor, arachidonic acid is catalyzed by cyclooxygenase (COX) to generate prostaglandin H2 (upregulated in serum), which is further converted into prostaglandin E1 (upregulated in ovary tissue) within the ovaries [22]. Unlike mammals, chicken ovulation may predominantly depend on prostaglandin E1 and its derivatives, including 1a,1b-dihomo prostaglandin E1, rather than PGE2 [23]. Prostaglandins promote angiogenesis around follicles by inducing vascular endothelial growth factor (VEGF) expression [24]. This vascular development supplies essential oxygen and nutrients to support rapid follicular growth [25,26]. Additionally, tissue accumulation of 15-deoxy-δ12,14-prostaglandin J2 likely enhances lipid droplet formation via PPARγ activation, reserving cholesterol esters for sustained steroid hormone synthesis [27,28]. These steroid hormones are indispensable for avian reproductive system development, particularly during growth stages. Notably, the significantly enriched steroid hormone biosynthesis pathway (map00140) further reveals the molecular characteristics of ovarian functional development. The dynamic changes of corticosterone and estrone in serum are stage-specific. The high expression of corticosterone in the early laying period may provide precursor reserves for the initial development of the ovary [29,30], while the high expression of estrone during the peak laying period directly supports estrogen synthesis required for follicle maturation [31]. The expression trend of estrone is consistent with the trend observed in serum hormone levels. The spatiotemporal distribution differences of these two metabolites suggest that poultry precisely regulate the balance of different hormone syntheses to meet the demands for reproductive hormones at different physiological stages.

### 4.3. Crosstalk Between ECM Precursors and Polyamines in Ovarian Development

During rapid ovarian development in chickens, extracellular matrix (ECM) remodeling is essential for follicular morphology maintenance and granulosa cell migration. Collagen produced by granulosa cells interacts with theca cells to promote follicular growth [32]. In this study, the upregulation of proline–hydroxyproline in serum indicates enhanced supply of collagen synthesis precursors. Proline, a major component of collagen [33], requires prolyl hydroxylases (PHDs) for hydroxylation [34], which utilizes α-ketoglutaric acid (serum↑) as a cofactor [35]. This metabolic coupling demonstrates that the ovary may utilize serum-derived α-ketoglutaric acid and proline to drive collagen fiber synthesis in the follicular membrane, providing mechanical support for development. Notably, although the arginine–proline metabolism pathway (map00330), which is closely related to collagen synthesis, was not significantly enriched, the coordinated changes in its key metabolites, spermine and L-arginine, suggest potential physiological regulatory pathways. L-arginine can be catalyzed by arginase (ARG) to convert into a proline precursor, further supporting collagen hydroxylation [36,37]. Meanwhile, as a substrate for polyamine synthesis (spermidine/spermine), L-arginine may enhance follicular microenvironment homeostasis through two pathways, ROS scavenging and AMPK/mTOR pathway activation. Tissue-elevated polyamines reduce oxidative stress via ROS scavenging and delay cellular senescence through AMPK/mTOR pathway-mediated autophagy [38,39]. These molecules also regulate cell proliferation and differentiation via the TGF-β/Smad pathway and modulate ECM components, enhancing cell matrix adhesion [40]. Based on our findings, we propose that during ovarian development, spermidine and spermine may protect follicular cells from oxidative damage through their antioxidant properties. Additionally, these polyamines might promote theca and granulosa cell proliferation by modulating the TGF-β/Smad signaling pathway. We further hypothesize that their effects on extracellular matrix remodeling could establish a stable microenvironment to support follicular growth. Collectively, these mechanisms are likely to drive rapid early ovarian development and enhance resistance to oxidative injury.

Characterizing the dynamic alterations in metabolites provides critical biological insights for phenotype interpretation, while comparative analysis of metabolic profiles across tissues reveals multiscale regulatory mechanisms from a system-level perspective [41]. The exchange of metabolites between tissues plays a pivotal role in systemic development, coordinating localized metabolic signaling with organismal demands to synchronize the optimization of tissue functionality and systemic maturation processes. During reproductive phases, the ovary as a key regulatory organ not only modulates its own metabolic homeostasis through microenvironmental regulation, but also integrates peripheral systems and interorgan metabolite crosstalk mechanisms to sustain its metabolic equilibrium [42,43].

While this study focused on metabolomic characterization of serum and ovarian tissues in a local chicken breed, the findings may provide valuable insights for commercial laying hens. Future investigations should prioritize multi-tissue metabolic mapping, potentially extending these analyses to commercial breeds to comprehensively delineate ovarian metabolic networks and their dynamic remodeling during reproductive processes. Such integrated approaches will elucidate critical mechanisms underlying folliculogenesis, ovulatory regulation, and steroid hormone biosynthesis, while identifying potential metabolic targets to enhance reproductive efficiency. Furthermore, future studies could incorporate tissue samples from yolk-removed hierarchical follicles across both early and peak laying phases. These analyses would help clarify the metabolic changes associated with follicular development at different stages of egg production. In addition, including specimens from hens exhibiting follicular maturation prior to their initial oviposition during early production periods may yield novel insights into the initiation mechanisms of reproductive cycles.

## 5. Conclusions

This study revealed stage-specific metabolic reprogramming in Taihe black-boned silky fowl through comparative metabolomic analysis of serum and ovarian tissues during the early laying stage and peak laying period. Ovarian tissues exhibited more pronounced metabolic changes compared to serum. Lipid metabolism emerged as a key driver, with prostaglandin E1 synthesis and arachidonic acid metabolism regulating ovulation and steroid hormone production, while FAHFAs enhanced antioxidant defenses during follicular development. Additionally, we propose that serum metabolites, such as spermidine and spermine, interact with ovarian pathways, including extracellular matrix remodeling, to coordinate cell adhesion, tissue remodeling, and oxidative stress resistance. These findings deepen our understanding of avian ovarian biology and provide actionable metabolic targets for improving poultry reproductive efficiency.

## Data Availability

All data presented in this article can be obtained by contacting the corresponding author.

## Supplementary Materials

The following supporting information can be downloaded at: https://www.mdpi.com/article/10.3390/ani15070912/s1. Supplementary Figure S1. Pearson correlation of quality control samples in metabolomics analysis; Supplementary Figure S2. Principal component analysis of metabolic profiles; Supplementary Figure S3. Pathway and classification annotations of serum metabolites; Supplementary Figure S4. Pathway and classification annotations of ovarian metabolites; Supplementary Table S1. Metabolites with significant expression differences in serum; Supplementary Table S2. Metabolites with significant expression differences in ovary; Supplementary Table S3. KEGG pathway enrichment analysis in serum; Supplementary Table S4. KEGG pathway enrichment analysis in ovary.

## Abbreviations

The following abbreviations are used in this manuscript:

20W	20-weeks-old
30W	30-weeks-old
COX	Cyclooxygenase
E2	Estradiol
ECM	Extracellular matrix
FSH	Follicle-stimulating hormone
LH	Luteinizing hormone
mTOR	Mammalian target of rapamycin
PBS	Phosphate-buffered saline
PRL	Prolactin
P4	Progesterone
TBSF	Taihe black-boned silky fowl
