# Supplementary figures and images for: Metabolic Profiles of Serum and Ovarian Tissue in Taihe Black-Boned Silky Fowl During the Early and Peak Laying Periods
Source: Animals (Basel). 2025 Mar 22;15(7):912. doi: 10.3390/ani15070912 (PMC11987729; doi:10.3390/ani15070912)

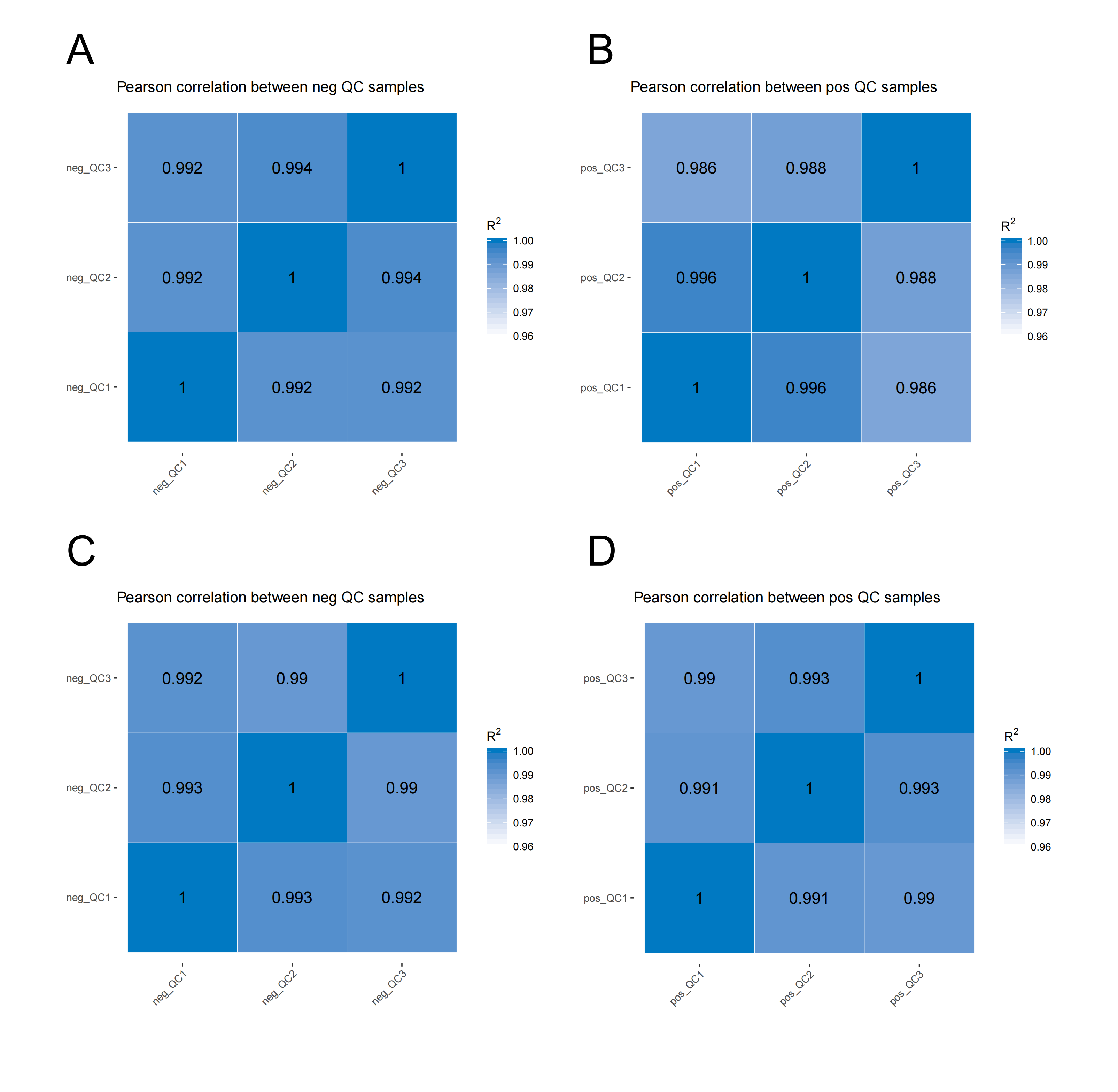

Supplement: Supplementary file 1 [file animals-15-00912-s001.zip › Supplementary Figure S1.png]

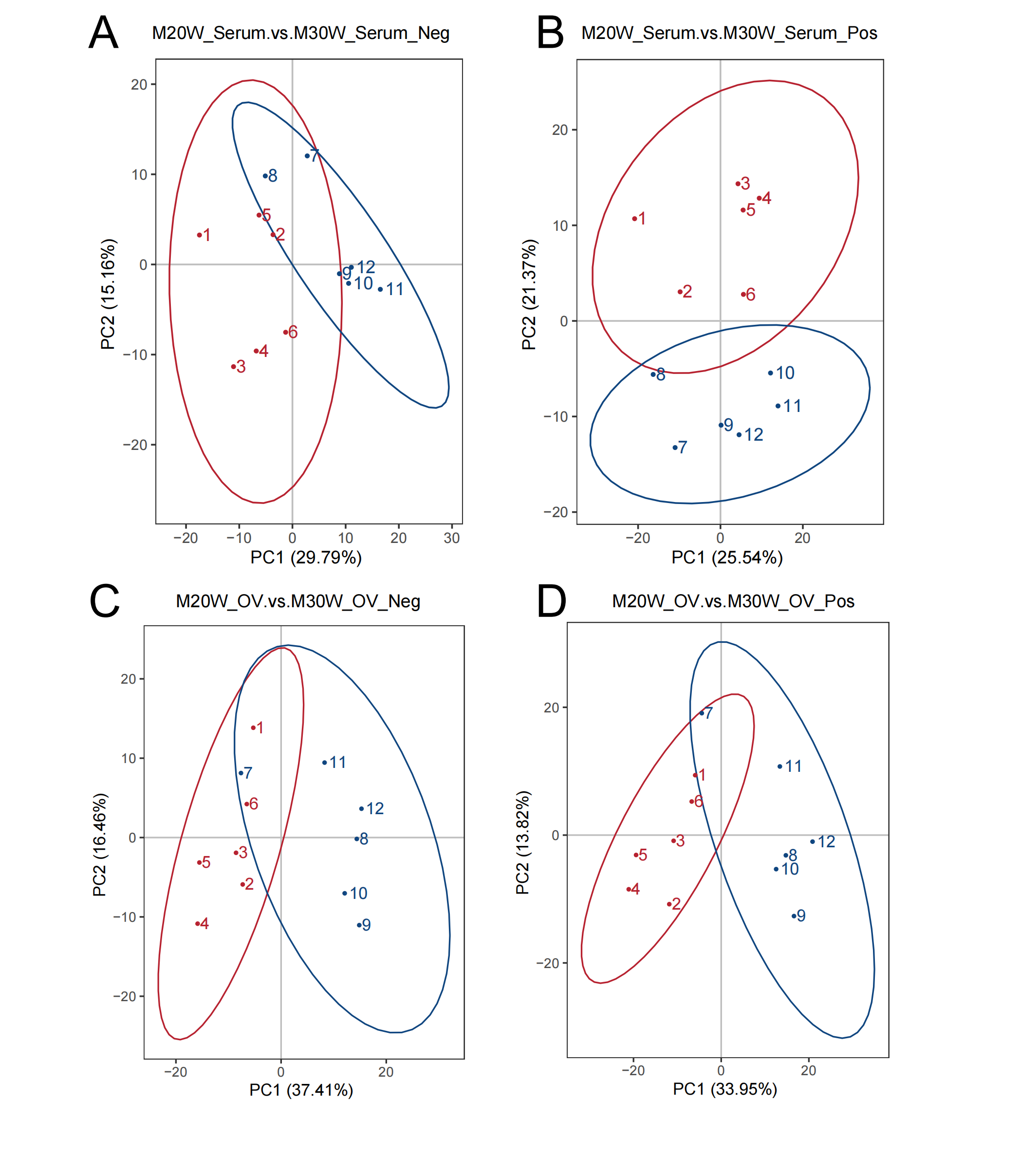

Supplement: Supplementary file 1 [file animals-15-00912-s001.zip › Supplementary Figure S2.png]

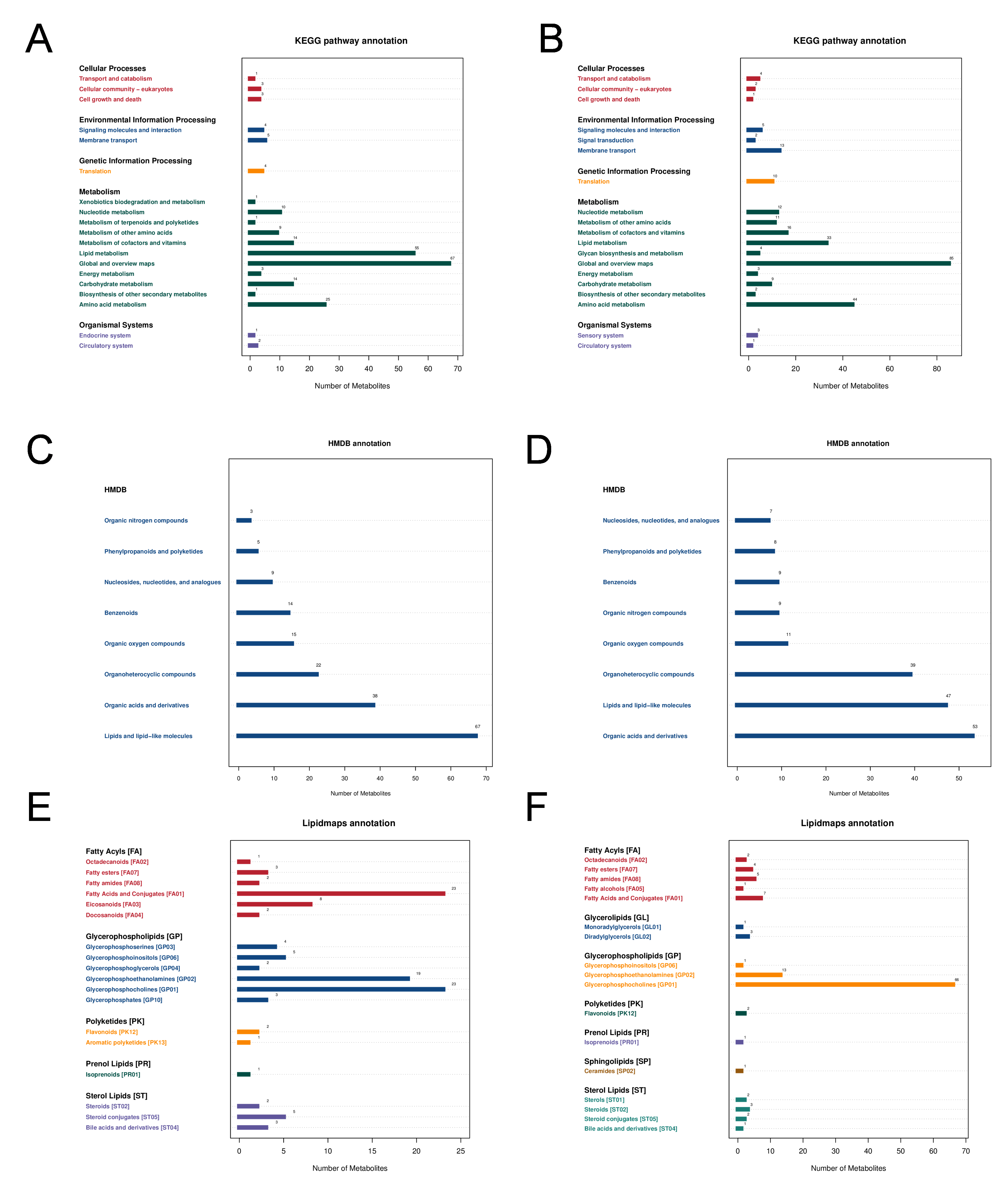

Supplement: Supplementary file 1 [file animals-15-00912-s001.zip › Supplementary Figure S3.png]

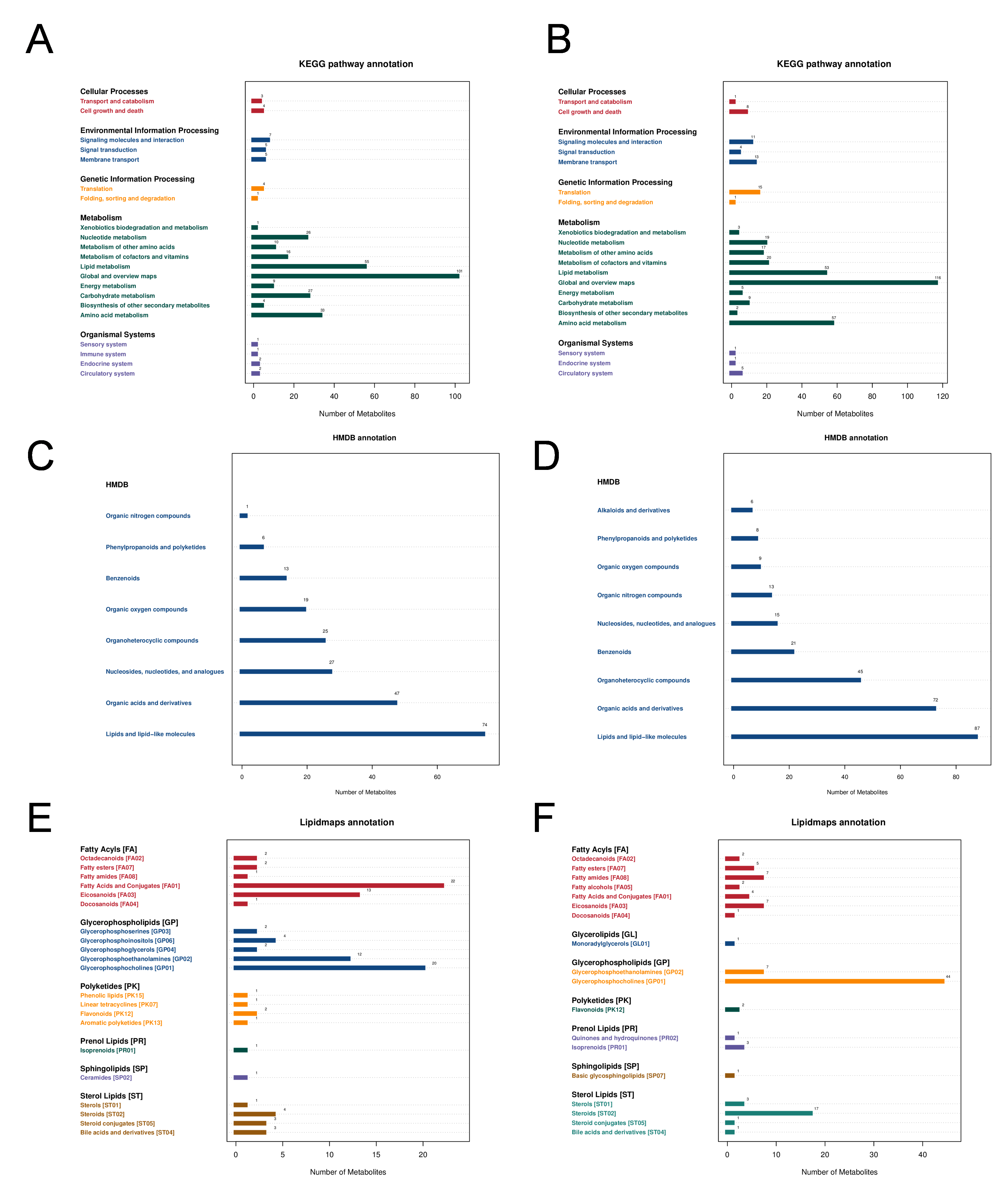

Supplement: Supplementary file 1 [file animals-15-00912-s001.zip › Supplementary Figure S4.png]
